# Acute Rheumatism

**Published:** 1892-06

**Authors:** 


### ACUTE RHEUMATISM.

℞ Euonymin, gr. 1-4.
Podophyllin, gr. 1-8.
Aloin, gr. 1-8.

M. Ft. pil. No. 1. Sig.—Give night and morning as necessary.

Give alkalies till saliva is alkaline. Following is a useful combination:

R Lithii benzoatis, dr. ss.

Sod. brom., } aa dr. ij.

Potas. carb., }

Potas. acet., oz. jss.

Sodii phos., oz. ss.

Syr. zingib., } aa ad oz. vj.

Aq. menth. pip., }

M. Sig.—Dr. ij to oz. ss in water, four to six hours after meals.

For antipyretics use antipyrin gr. x and digitalis gr. j, combined. For analgesics use phenacetin or antipyrin, and, if necessary, a combination of morphine, bromide, and chloral. Give alkaline mineral waters copiously. Give tonics, the following being excellent:

R Tr. ferri chlor., dr. iv.

Tr. nuc. vom., } aa dr. ij.

Ac. phos. dil, }

Syr. aurantii cort., oz. j.

Elix. calisaya, q. s. oz. ij.

M. Sig.—Oz. j in water t. i. d. before meals.

If heart weakens, use following:

R Spts. ammon. aromat., oz. iij.
Ammon. carb., dr. j.
Tr. cardomom. oz. j.
Tr. nuc. vom., dr. iij.

M. Sig.—dr. j t. i. d.

For topical application use:

R Tr. aconit. rad., } aa oz. ss.

Tr. arnica, }

Chloroform, oz. j.

Tr. saponis camph., oz. ij.

M. Sig.—Apply locally.

Exclude nitrogenous foods. Salicylates, baths, and massage are of doubtful value.—F. Le Roy Satterlee, M. D.—*Med. Age, April 25, 1892, p. 255.*

